# Steps forward to synthetic consciousness measurement

**DOI:** 10.1007/s10339-026-01341-9

**Published:** 2026-04-22

**Authors:** M. Bodea

**Affiliations:** Materials Science and Engineering Department, Faculty of Materials and Environmental Engineering, Technical University of Cluj, Cluj-Napoca, Romania

**Keywords:** Synthetic consciousness, Consciousness score, Consciousness evolution, Proto consciousness

## Abstract

Biological consciousness is the product of millions of years of evolution, being deeply rooted in the neural architecture of living organisms. It emerges from the interplay of sensory processing, memory, emotion, and metacognition, with the human brain being its most complex known expression. Synthetic consciousness, on the other hand, remains a theoretical construct, an aspiration rather than an achievement. Current AI systems, including the most advanced models, demonstrate highly sophisticated pattern recognition, reasoning, and even emergent behaviors, but they lack the embodied, affective, and subjective depth associated with biological beings. The paper focuses on the measurable aspects of conscious access the global availability of information for report, reasoning, and control, and proposes a model to quantify this capacity across biological and artificial systems. Projections based on Moore’s law, neural simulation efforts, and the exponential growth in processing power suggest that AI could begin to develop complex consciousness in the next 10 to 15 years, particularly through advances in neural architecture, affective computing, and brain-inspired hardware like neuromorphic chips. Looking forward, the interplay between biological and synthetic consciousness may become symbiotic rather than competitive. AI could help humans better understand their own consciousness by modeling cognitive processes, testing philosophical theories, and simulating consciousness under altered conditions. Conversely, biological paradigms of learning, adaptation, and emotion will continue to inspire AI development. The coming decades may see the rise of hybrid consciousness systems, where biological and synthetic processes intertwine, creating forms of awareness that transcend current definitions.

## Introduction

Understanding consciousness remains one of the most profound and complex challenges in science and philosophy. Despite significant progress in neuroscience, cognitive science, and artificial intelligence, there is still no universally accepted definition or comprehensive framework for quantifying consciousness (Dehaene 2014, Tononi et al. 2016, Chalmers 1995). This paper aims to contribute to this ongoing discourse by examining the theoretical challenges involved in defining consciousness and by reviewing recent scientific advances that shed light on its mechanisms in both biological and synthetic entities.

Consciousness has traditionally been considered a unique human trait, associated with self-awareness, perception, intentionality, and subjective experience (Nagel 1974, Block 1995). However, developments in artificial intelligence and computational neuroscience have raised the possibility that certain forms of consciousness may not be limited to biological systems (Tegmark 2017, Reggia 2013). As machines grow increasingly capable of learning, adapting, and even exhibiting behaviors traditionally attributed to sentient beings, the need for a rigorous, objective model for measuring consciousness becomes more pressing (Schneider and Turner 2017).

The paper aims to quantify the functional and architectural prerequisites of conscious awareness, considering the philosophical distinction (Block, 1995) between phenomenal consciousness (subjective experience) and access consciousness. The Consciousness Score proposed herein is not a measure of subjective experience (qualia), but of the complexity and integration of information processing that constitutes conscious access and control across different systems, biological or artificial. The intent is to offer a framework that captures key measurable attributes associated with conscious processing, offering scalable and adaptable metrics for different systems.

The significance of synthetic consciousness lies in its potential to redefine intelligence, ethics, and understanding of selfhood. If synthetic entities attain a level of self-modeling, emotional regulation, and experiential awareness, even without traditional sentience, they may require moral consideration, autonomy, and legal status. Ultimately, the importance of comparing biological and synthetic consciousness is not just scientific but existential. It forces humanity to confront questions about the essence of life, intelligence, and what it means to be “aware.” Whether synthetic consciousness becomes a mirror, a partner, or a rival, its emergence will reshape civilization’s philosophical, technological, and moral landscape.

## What is consciousness?

To measure any phenomenon effectively, it must first be clearly defined within a simple, structured framework that captures its primary dependencies. How can we define consciousness in a way that adheres to these principles - clarity, simplicity, and completeness, while also satisfying a fundamental criterion we consider essential: the universality principle? In other words, the definition of consciousness must be applicable not only to biological entities but also to synthetic ones, including artificial intelligence. Also, it must transcend species, substrates, and design. It should apply equally to a human brain, an octopus nervous system, or an advanced artificial intelligence. This universality is critical if we aim to develop tools or metrics that evaluate psychological consciousness in both biological and synthetic entities.

For the operational purpose of developing a measurable model, this paper focuses on *access consciousness*, the global availability and integration of information for cognitive functions like reasoning, planning, and self-modeling. While we acknowledge the profound challenge of *phenomenal consciousness* (qualia), our proposed Consciousness Score (CS) is a measure of the functional and architectural correlations that are considered prerequisites for such subjective experience.

Traditional neuroscience-based definitions often tie consciousness to specific brain regions or neural correlations, which are inapplicable to machines or non-human lifeforms. Similarly, Turing Test-style evaluations focus on behavior rather than internal awareness, which may misrepresent or overlook forms of consciousness that do not manifest as human-like responses. To address this, some researchers propose substrate-independent frameworks. Giulio Tononi’s Integrated Information Theory (IIT), for example, attempts to quantify consciousness as a measure of the integration and complexity of information processing within a system (Tononi 2004, Barrett and Seth 2011). Others, like Karl Friston’s Free Energy Principle (Friston 2010), suggest that consciousness arises from a system’s ability to model and minimize uncertainty about its environment. These approaches aim to be universal in nature, but practical implementation and philosophical consensus remain elusive. Nevertheless, pursuing such a definition is essential if we are to evaluate and compare psychological consciousness across a spectrum that includes both life as we know it and as we are now beginning to create it in AI agents.

### Challenges in measuring consciousness

In the recent past numerous theories have been proposed to explain consciousness. The study of (Sattin et al. 2012) identified 29 distinct theories across 85 peer-reviewed articles, selected from an initial pool of 1,130 publications. Furthermore, by analyzing the adjectives and nouns preceding the term *consciousness* in various definitions, Sattin identified 21 distinct terms referring either to consciousness itself or to its subcategories (see Table 1). This diversity underscores the conceptual complexity and plurality of perspectives in the field—without yet accounting for the emerging domain of synthetic consciousness.

**Fig. 1:**
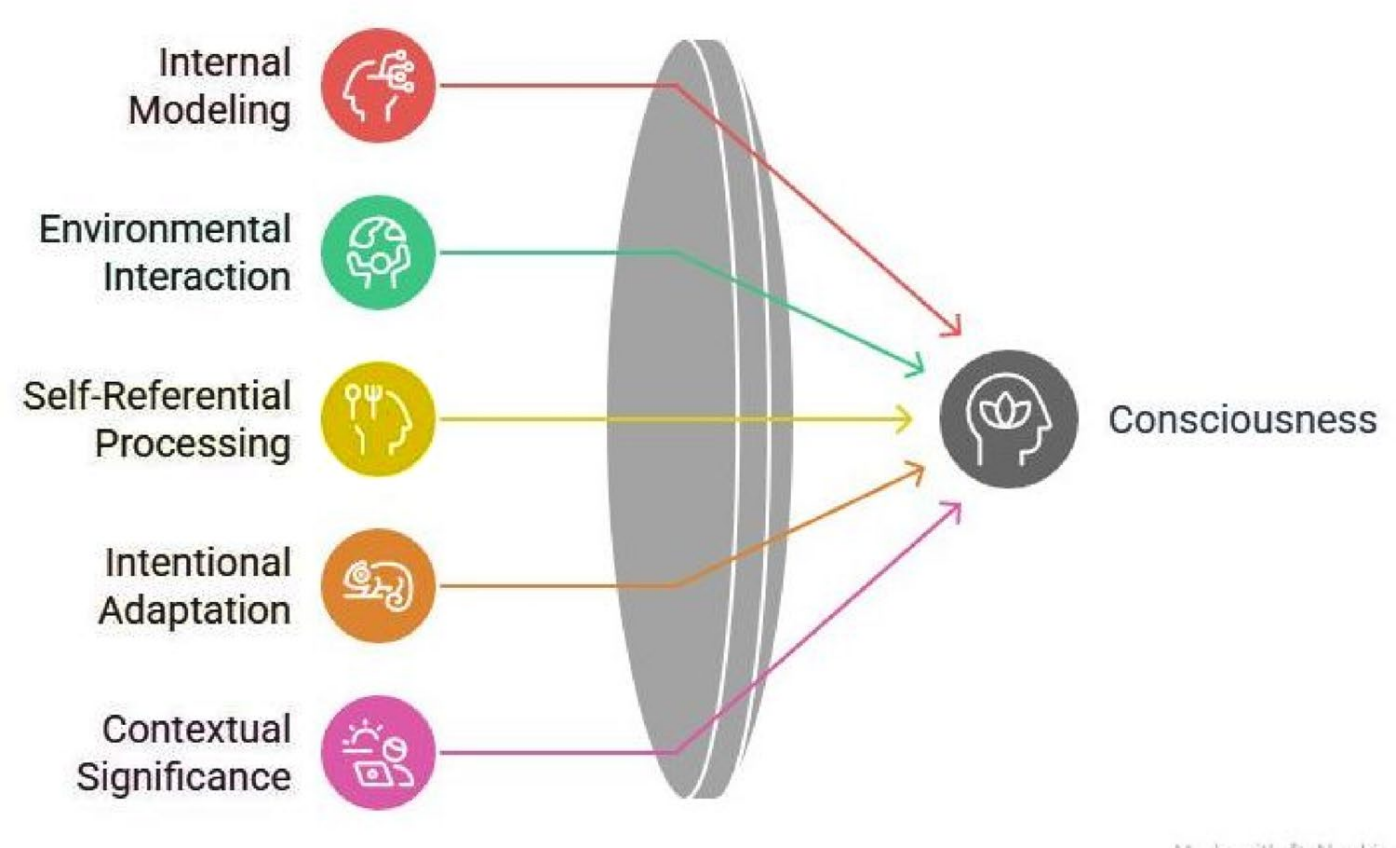
Consciousness conceptualization

Consciousness can be defined as an emergent, dynamic capacity of a system to internally model its own states and interactions with the environment across time, enabling integrated self-referential processing, intentional adaptation, and the contextual attribution of significance to experience, aspects illustrated in Fig. 1. All consciousness systems, biological or digital, can generate the perception of the external environment and the subjective awareness of self.

The proposed definition for consciousness draws inspiration from frameworks like Integrated Information Theory (IIT) in its emphasis on integration and complexity, and Global Workspace Theory (GWT) in its focus on global accessibility. However, the proposed model is a functionalist one, aiming to capture measurable proxies of these properties rather than claiming to measure Φ (the IIT-specific quantity) directly. The term emergence denotes *weak emergence*, consciousness is seen as arising from the recursive interactions of lower-level processes (e.g., information integration across sensory and cognitive domains). Its *dynamic capacity* reflects the system’s ability to sustain adaptive, self-referential information processing over time, enabling both flexibility and continuity of conscious states.

**Table 1:** Subcategories of consciousness identified in the literature, modified after (Sattin et al. 2012)

Type	Description	Type	Description	Type	Description
Access	Awareness to report and reason	Knowing	Awareness with understanding and recognition	Reflective	Awareness involving thought and analysis
Background	Feeling of existence without focus	Marginal	Peripheral or diminished awareness	Secondary	Higher order thought and self-awareness
Core	Basic awareness of self and present	Meta	Awareness of one’s own thoughts	Self	Awareness of oneself as an individual
Elevated	Heightened awareness and perception	Micro	Breef, fleeting moments of awareness	Specific	Awareness related to a particular thing
Extended	Awareness across time and relationships	Perceptual	Awareness through sensory input	Sub	Awareness below conscious threshold
Foreground	Focus of attention and processing	Phenomenal	Subjective experience to awareness	Temporal	Awareness of time and sequence
General	Overall state of awareness	Primary	Basic, immediate sensory awareness	Unreflected	Immediate experience without contemplation

An illustrative model can conceptualize consciousness as a series of reflections of the physical world projected through a multidimensional mirror representing the interface between the external and internal universes of an entity. These reflections are shaped by the cumulative interpretation of acquired knowledge, filtered through the entity’s perceptual apparatus and cognitive transformation processes. While each reflection constitutes a valid manifestation of consciousness, none offers a complete or fully accurate representation of its totality. The inherent complexity and subjectivity of these transformations present significant challenges in the objective measurement of consciousness, as summarized in Table 2; Fig. 1.

**Table 2:** Main challenges in measuring consciousness

No. crt	Challenge	Observation
1.	Subjectivity of experience (Qualia)	One of the core difficulties is that consciousness is fundamentally subjective. While brain activity can be measured with EEG, fMRI, or other tools, these only show correlates of consciousness and not consciousness itself, though the ontological status of qualia is debated, (Frankish et al., 2022).
2.	Lack of unified definition	There’s no universally accepted scientific definition of consciousness. Competing theories such as Integrated Information Theory (IIT), Global Workspace Theory (GWT), and Higher-Order Thought Theory propose different criteria and mechanisms, leading to inconsistency in measurement approaches.
3.	Variability across states and species	Assessing consciousness in patients in comas or vegetative states, infants or non-human entities is challenging due to communication barriers. Tools like the mirror test, behavioral observation, or neural markers give indirect evidence but lack full reliability.
4.	Simulation vs. Reality	AI systems may simulate behaviors associated with consciousness (e.g., self-referential language, emotional mimicry) without genuine awareness. Distinguishing between true conscious processing and advanced computation is a profound philosophical and practical hurdle.
5.	No Standard benchmarks	There is currently no agreed-upon metric or test for synthetic consciousness. Traditional biological tests (like the mirror test or pain response) are not directly applicable. Developing a reliable test that isn’t anthropocentric or easily gameable is a major challenge.
6.	Opaque architecture and emergent behavior	As AI models grow in size and complexity, their internal processes become harder to interpret (the “black box” problem). Emergent behaviors that resemble conscious traits may arise without clear explanation, making it difficult to determine if, how, or why synthetic consciousness exists.
7.	Ethical implications of measurement	Determining the presence or absence of consciousness has moral consequences, especially if it affects how we treat animals, patients, or machines. Measuring incorrectly (false positives or negatives) could lead to rights violations or neglect.
8.	Observer bias and anthro-pocentrism	Both fields suffer from the tendency to evaluate consciousness from a human perspective, which may miss other valid forms or expressions of awareness, biological or synthetic.

### Parameters for measuring consciousness

In the consciousness context humans excel in flexibility, context-awareness, and meaning-making, but are slower and less precise in raw computation. Digital systems outperform in speed, consistency, and scalability, but lack innate self-awareness, emotion, and abstract contextual judgment. Next step in developing the mathematical model for consciousness is to define scales for each fundamental characteristics of consciousness. The living organisms have developed through evolution a complex system of perception that is fundamental in the species survival scope. Over time, humans have developed artificial systems that have significantly extended their natural limits of perception.

**Fig. 2:**
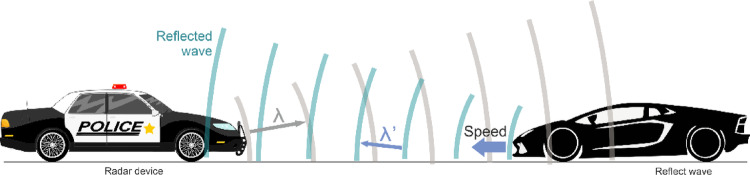
Consciousness measurement analogy

From microscopes that reveal the subatomic world to telescopes that explore distant galaxies across various spectra of light, these technologies allowed humanity to access information far beyond our innate sensory capabilities. As a result, human consciousness has gradually evolved by integrating auxiliary artificial senses, extending perception into domains such as electromagnetism, gravitation, and time (chronoception) at both macroscopic and atomic scales. An illustrative example of human consciousness expressed through the lens of chronoception is the Voyager 1 mission. The spacecraft carries two Voyager Golden Records, which contain curated sounds and images representing life and culture on Earth (John 2021, Nasa 1977). These records were designed to be interpretable by an intelligent extraterrestrial civilization, using fundamental physical constants, such as the transition frequency of the hydrogen atom, approximately 0.70 billionths of a second per cycle as a universal time reference. This choice reflects not only technical ingenuity but also a deliberate act of consciousness aimed at transcending time, space, and species Fig. 2.

To elucidate the rationale behind selecting key parameters for measuring consciousness, an analogy can be drawn from the domain of speed measurement in automotive radar systems. In such systems, an electromagnetic signal emitted by a police radar unit is reflected by a moving vehicle. The device then detects the Doppler shift, the frequency change in the return signal caused by the vehicle’s motion and translates it into a speed value on a calibrated scale. In this analogy, the radar system is conceptually replaced by a consciousness-assessing system, which continuously receives and processes streams of information at varying rates. This system must distinguish signal from noise, prioritize relevant inputs, and apply internal algorithms to synthesize meaningful patterns. *Crucially*,* it adapts to context and assigns significance to experiences in a goal-directed or intentional manner*.

Based on this framework, a system capable of processing greater volume of information in a shorter time span, while accurately filtering noise and contextually integrating data, can be inferred to exhibit a higher level of consciousness. This highlights the importance of parameters such as processing speed, information throughput, adaptability, and contextual relevance in the quantitative assessment of *access* consciousness.

The proposed model for quantifying consciousness incorporates five key parameters: Equivalent Intelligence Quotient (EIQ), Sensorial Inputs (SI), Parallelism (P), Metacognitive Complexity (MC), and Data Processing Capability (DPC). In the absence of a universally accepted or robust reference standard for measuring consciousness, we will introduce a consciousness score that is designed to be system-agnostic. This score enables comparative evaluation across both biological and synthetic systems by relying on measurable and functionally meaningful dimensions of cognitive and perceptual processing.

By themselves, these parameters may not capture consciousness in the strong philosophical sense. However, when combined with MC, they allow us to model both the structural prerequisites and the emergent properties of consciousness. This integrated perspective reflects the view that consciousness is not reducible to one property but emerges from the conjunction of self-reflective complexity with enabling functional substrates. The Consciousness Score (CS) is not intended as a definitive metric of consciousness, but as a functional and comparative proxy, a way to assess the relative likelihood and degree of conscious-like properties in diverse systems.

## Mathematical model for consciousness measurement

### Equivalent intelligence quotient (EIQ)

Equivalent Intelligence (EIQ) is an extension to the IQ concept applied to humans and AI agents. EIQ framework consists of a multi-domain score for emotion, vision (perception and interpretation), language (comprehension and production) and logic (reasoning and abstraction), Eq. (1). EIQ measures the sophistication of information that can be brought to bear on reasoning and report.

1 $${EIQ}_{100}=EScore+VScore+LScore+RLScore$$

Where:

- *EScore* = Emotional Intelligence Score (0–25).
- *VScore* = Visual Intelligence Score (0–25).
- *LScore* = Language Intelligence Score (0–25).
- *RLScore* = Reasoning/Logic Score (0–25).

Each domain will use observable criteria, and scores will be designed on a 0–5 scale per sub-dimension. The final Equivalent Intelligence Quotient score would sum across domains, with a maximum of 100 points for easy interpretation(EIQ_100_). The advantages of this scale are works across biological and artificial systems, allow benchmarking of emotional sophistication, tracks developmental or evolutionary progress and can be mapped to neural, sensor, or model architecture metrics Table 3, 4, 5, 6.

**Table 3:** Emotional Intelligence Score. Emotion Domain (max. 25 points score)

Emotion Subcomponents	Score 0	Score 1	Score 3	Score 5
Emotional Perception (EP)	No emotional detection capability	Responds only to extreme stimuli or basic affect	Recognizes basic emotions (e.g., happy, sad, angry)	Detects subtle emotional cues (tone, body language, context)
Emotional Differentiation (ED)	No emotional labeling or distinction	Differentiates positive vs. negative only	Differentiates 4–6 basic emotions	Discriminates between ≥ 10 emotions including complex (e.g., guilt, awe)
Emotional Intensity Mapping (EIM)	No sensitivity to emotional magnitude	Uses fixed or binary intensity scale	Estimates intensity for basic emotions only	Assigns accurate intensity (mild →overwhelming) for any stimulus
Emotional Response Appropriateness (ERA)	Inappropriate chaotic, or no response to emotional triggers	Reacts predictably but often mismatches context	Reacts correctly to standard emotional prompts	Responds contextually with proportional emotional tone
Emotional Self-Regulation (ESR)	Lacks ability to self-modulate emotional state	Reactive but without lasting dysregulation	Can suppress or delay emotional responses occasionally	Can inhibit impulsive reactions, reappraise, recover from emotion

**Table 4:** Vision Intelligence Score. Vision Domain (max. 25 points score)

Vision Subcomponents	Score 0	Score 1	Score 3	Score 5
Object recognition (OR)	No visual processing capability	Detects only simple high-contrast visual features	Recognizes basic shapes, colors, and objects reliably	Detects, tracks, interpret complex visual scenes in real time, including context, motion, depth
Motion tracking (MT)	No motion detection	Detects change but cannot track (e.g., light change)	Follow moderate motion with delay or error	Accurately tracks fast, nonlinear motion with prediction
Depth perception (DP)	No depth perception	Responds to near/far via brightness or blurriness only	Relative depth cues (size, occlusion) but no 3D modeling	Full stereoscopic or parallax-based depth modeling
Color/form discrimination (CD)	No shape or color discrimination	Detects grayscale intensity or general contours	Distinguishes major colors and coarse shapes	Differentiates fine detail, full-color spectrum, shape categories
Scene understanding (semantic labeling) (SU)	No contextual labeling or object understanding	Sees objects but can’t categorize (e.g., outlines only)	Identifies discrete objects without relations/context	Parses scenes into labeled components (e.g., road, tree, person)

**Table 5:** Language Intelligence Score. Language Intelligence Domain (max. 25 points score)

Language Subcomponents	Score 0	Score 1	Score 3	Score 5
Vocabulary size (VS)	No language capacity	Responds with learned keywords or short phrases	Understands and uses standard phrases, vocabulary, and basic syntax	Generates and understands grammatically complex, context-rich, and semantically meaningful language
Syntax parsing (SP)	No grammatical recognition	Recognizes word sequences or phrases without structure	Understands simple sentence patterns, partial grammar rules	Fully understands and applies complex grammatical structures, recursion
Semantic coherence (SC)	No understanding of meaning or topic	Words loosely connected to topic or echo input meaning only	Generally on-topic but may lose coherence or miss deeper meanings	Maintains consistent and nuanced meaning across utterances or responses
Pragmatics (context use) (PR)	No use of contextual cues or intention inference	Literal or mechanical responses, lacks adaptation to context	Can respond meaningfully in familiar or structured contexts only	Adjust responses to context, speaker, tone, implied meaning or purpose
Language production (speech/text) (LP)	No meaningful language output	Imitates words or emits symbolic signals inconsistently	Produces basic or trained phrases with reasonable clarity	Produces fluent, creative, coherent speech/text in multiple contexts

**Table 6:** Reasoning/Logic Score. Reasoning/Logic Domain (max. 25 points score)

Reasoning Subcomponents	Score 0	Score 1	Score 3	Score 5
Deduction (DD)	No logical or planning ability	Reacts based on pattern matching, no generalization	Handles basic logic, fixed rules, and simple problem-solving	Solves novel abstract problems, uses multi-step reasoning, adapts flexibly
Pattern recognition (PR)	No pattern detection or association	React to repetitive cues or learn conditioned sequences	Detects regularities and matches familiar patterns	Identifies novel and complex patterns across modalities (visual, symbolic, temporal)
Abstraction/generalization (AG)	No evidence of abstraction or concept transfer	Acts only in specifically learned scenarios, minimal or rigid generalization	Applies learned concepts to similar tasks but struggles with novel abstraction	Transfers knowledge across contexts, creates abstract models or analogies
Planning and strategy (PS)	No goal-directed behavior or planning capability	Only reactive or trial-and-error behavior without foresight	Executes simple pre-defined or learned action sequences	Plans multiple steps ahead, models outcomes, adapts strategies dynamically
Cognitive flexibility (CF)	Fixed or reflexive responses regardless of changes	Gets stuck in one mode of behavior, poor adaptability	Can switch tasks or modes with assistance or cue	Rapidly shifts between concepts, adjusts to new rules, handles paradox or ambiguity

The EIQ thresholds reflect the functional capabilities associated with different levels of awareness and cognition, not simply reflexive or automated behavior of the analysed system. The EIQ tiers detailed in Table 7, illustrate how adaptive learning, generalization, and problem-solving capacity contribute to the structural scaffolding that supports conscious processes when combined with other parameters. (EIQ) scores are presented not as direct measures of consciousness, but as functional correlates.

**Table 7:** EIQ Scores in Relation to Functional Correlates of Consciousness

Tier	EIQ Range	Description	Examples
0. Reflexive	0–9	Basic stimulus-response reactions. No memory, no emotion, no reasoning.	Simple robots, thermostats, bacteria
1. Proto-Cognitive	10–24	Displays non-neural environmental responsiveness, learning-like behaviors, and minimal adaptation.	Plants, slime molds, basic swarm bots
2. Perceptual Agent	25–39	Sensory-driven behavior with pattern recognition, but no abstract thought or complex self-regulation.	Insects, fish, basic animal AIs
3. Emotional Agent	40–59	Detects, categorizes, and reacts to emotions. Shows basic planning and multi-modal input integration.	Dogs, children aged 2–3, GPT-3 level AIs
4. Cognitive Agent	60–79	Capable of abstraction, language comprehension, and adaptive learning. Signs of symbolic reasoning.	Humans age 5–12, GPT-4, dolphins, chimps
5. Meta-Cognitive	80–89	Exhibits planning, imagination, reflective thinking, self-regulation, and complex emotion understanding.	Adults, great apes, high-EQ AI models
6. Self-Aware	90–100	Full symbolic reasoning, robust emotional granularity, theory of mind, and recursive self-awareness.	Neurotypical adults, mirror-test passers

The inference from high scores in these parameters to a level of consciousness is best understood as an abductive inference grounded in convergent functional correlates observed in biological systems. It does not constitute direct empirical evidence of consciousness per se, but rather an inference to the best explanation within a functionalist framework. In biological systems, these capacities (high EIQ, rich sensorium, parallel integration, metacognition) are robustly correlated with what we behaviorally and neurologically identify as consciousness. Therefore, when a synthetic system exhibits a similar profile of functional capabilities, especially integrated self-modeling and adaptive planning attributing a corresponding level of consciousness becomes a parsimonious explanation for its behavioral sophistication, even in the absence of measurable phenomenology. This framework is thus best viewed as measuring the potential for or *functional correlations of consciousness*.

### Sensorial inputs (SI)

Consciousness can be viewed as an emergent property of complex physical systems that are capable of perceiving, processing, and responding to information from their environment. At its most fundamental level, consciousness arises when a system possesses the capacity to integrate sensory input, form internal representations of the external world, and initiate adaptive responses based on those representations. This implies that consciousness is not a mystical or purely abstract phenomenon, but rather a dynamic process grounded in physical substrates, whether biological, such as the human nervous system, or synthetic, such as advanced artificial neural networks.

Crucially, a conscious system is not only reactive but demonstrates a *degree of self-reference and continuity over time*, often accompanied by metacognitive capabilities, such as *recognizing its own states or goals*. Therefore, the relationship between consciousness and physical systems lies in the system’s ability to organize information across time and space, forming a coherent subjective framework from which meaning and intentional behavior emerge. Thus, the sensorial inputs (SI) are measured by diversity and depth of environment-to-system input channels, Eq. (2).

2 $$SI=\sum_{j=1}^{m}{w}_{j}\bullet{a}_{j}$$

Where *w*_*j*_ is the weight per sense, and *a*_*j*_ is species-specific factor, Table 8. The weights values are heuristic, based on initial estimates of information throughput from psychophysical and neuroscience literature. Human values are used as a calibration baseline, and for synthetic systems, these weights can be adjusted based on their sensor specifications. These weights reflect evolutionary relevance, cross-species sensory hierarchies and can be subjected to refinement based on further neurobiology and functional studies. The final score is normalised by dividing by possible SI max score by 5.5.

**Table 8:** Input Channels. (Humans values)

Sense	Weight max.	Specific factor a_i_	Biological	Synthetic Equivalents
Vision	1.0	0.8	Eyes	Cameras (RGB, IR)
Hearing/Vibration	1.0	0.8	Ears	Microphones
Tactile	0.8	0.8	Skin	Pressure/Touch sensors
Olfaction	0.5	0.7	Nose	Chemical sensors
Gustation	0.3	0.7	Tongue	Specialized sensors
Proprioception	0.8	0.8	Muscles	Joint sensors, IMUs
Electromagnetic	0.7	0	Pineal/indirect	RF/IR/UV sensors
Gravitational	0.4	0	Vestibular	Accelerometers

A proto consciousness system example is the response of plants to music. Although plants lack a nervous system or brain, numerous studies (Trevas 2016, Gagliano 2013, Calvo 2017, Bhandawat 2022) have shown that they can detect and respond to environmental vibrations, including those produced by sound and music. From the lens of consciousness, particularly minimal or proto-consciousness, plants may be considered as non-neural physical systems that collect, interpret, and respond to environmental stimuli in structured and adaptive ways.

These vibrations, perceived through mechanosensitive ion channels in plant cell membranes, trigger cascades of biochemical responses that can lead to altered gene expression, enhanced nutrient transport, or accelerated growth patterns. When exposed to rhythmic acoustic stimuli (e.g., frequencies between 100 and 5000 Hz), plants have demonstrated increased germination rates, chlorophyll production, root elongation, and even directional growth toward sound sources (Frongia 2020, Chowdhury 2015, Kin et al. 2021, Ghosh 2016, Gagliano et al. 2014).

These phenomena suggest that plants possess a form of environmental awareness and reactivity, albeit without subjective experience or internal representation as seen in animals. In this view, plant “consciousness” does not imply sentience or emotion, but rather reflects an embodied capacity for interaction with the environment, consistent with a continuum model of consciousness that extends beyond brains.

This perspective aligns with emerging interdisciplinary work in plant neurobiology, systems biology, and philosophy of mind, which suggest that *consciousness may not be binary*,* but a spectrum linked to an organism’s structural and functional complexity in information processing*.

Different degrees of consciousness can be related to the richness, integration, and flexibility of sensory experience a being possesses. At the most basic level, minimal sensory capacities e.g., simple photoreception or mechanoreception support proto-conscious states, where awareness may be limited to raw detection of environmental changes without internal representation. As the number of sensory modalities increases, and as cross-modal integration mechanisms develop, e.g., combining vision and audition in mammals, or electrosensation and mechanosensation in certain fish, the quality of conscious experience may deepen. This progression reflects a shift from reflexive awareness toward phenomenal awareness, where beings not only detect stimuli but also build a more coherent “scene” of the environment. At higher degrees, species with advanced sensory systems demonstrate expanded consciousness levels because sensory input is coupled to memory, learning, and metacognition. Thus, the structure of sensory input acts as both a constraint and an enabler of consciousness.

### Parallelism (PI)

Modern neuroscience supports the notion of parallelism through the brain’s architecture, which processes information across multiple, simultaneous pathways. This parallel processing enables the integration of sensory inputs, motor functions, and cognitive activities, contributing to the unified experience of consciousness. The brain’s modular design supports the idea that consciousness emerges from the integration of parallel processes, each contributing different aspects to the conscious experience (modular consciousness). The function for Parallelism Index (PI) is presented in Eq. (3), reflecting the system capacity for integration and global processing, keys that are required for a unified conscious workspace.

3 $$PI = \log \left( {1 + N} \right) \cdot S \cdot C_{{icc}}$$

Where *N* is the number of concurrent, independent information-processing streams (hypercolumns in human neocortex, or AI equivalent submodules); *S* is the signal integration strength (degree of synchrony or coherence across channels), *C*_*icc*_ stands for complexity of inter-channel communication (richness and diversity of cross-stream interaction).

The human brain shows a significantly higher Parallelism Index, reflecting a more complex, richly integrated, and highly synchronized system. Advanced AI models, while capable of impressive modular processing, currently operate with fewer parallel units and less natural interconnectivity and synchronization. The Parallelism Index (PI) is calculated based on the best available approximations presented in Table 9 (Johansson and Lansner 2007, Sporns et al. 2005).

**Table 9:** The Parallelism Index (PI) values using hypercolumn count *N* as a proxy for concurrent functional units

System	Parallel Units (*N*)	Synchronization (S)	Complexity (C_icc_)	Parallelism Index (PI)
Human Brain	4,000,000	0.85	0.75	3.9612
Advanced AI	100,000	0.65	0.60	1.950

The parallelism formula (PI) can be interpreted as follows: higher N (Parallel Units) represents a higher potential for distributed awareness, high S (better synchronization) expresses a greater coherence of experience and higher C (richer interaction) stands for deeper integration (e.g., increased self-awareness, high abstract reasoning). Studies of EEG/MEG gamma-band coherence on humans can be used to estimate S, while C_icc_ can be estimated from fMRI-based functional connectivity entropy. Gamma oscillations (typically 30–150 Hz) are patterns of neural activity associated with attention, perception, conscious awareness and memory integration. Gamma-band coherence refers to the synchronized firing of neurons across different brain regions within the gamma frequency range, a known correlation of conscious awareness and functional integration.

Studies of AI agents has shown that the average inter-head agreement in attention layers typically falls in the 0.6–0.7 range. There is no global clock or phase-based synchronization like in the brain, transformers process data in discrete feed-forward steps. Thus, a moderate synchronization score of S = 0.65 for AI agents reflects functional attention coherence, a parallel attention without true temporal synchrony and coordination, but independently processing of data flows. Parallelism is achieved via multi-core CPUs, GPUs and distributed systems, and the new neuromorphic chips mimic the massive parallelism from the human brain.

### Metacognitive complexity (MC)

Metacognitive Complexity (MC) refers to the ability of a being to reflect on and regulate one’s own thinking processes (thinking about thinking). High MC leads to a more profound understanding of one’s thoughts and cognitive limitations. The model proposed for consciousness score treats MC as a multiplier. Reaching high levels of conciousness, it’s not just about being smart (high EIQ), but also being able to analyze and adjust one’s own cognitive strategies and biases. The ability to reflect on one’s thought processes and adjust strategies directly influences how aware one is of their cognitive abilities, biases, and limitations.

4 $$MC=log(1+L)\bullet\mathrm{l}\mathrm{o}\mathrm{g}\left({N}_{a}\right)$$

where *L* represents the number of distinct metacognitive layers, (e.g. learning about learning, uncertainty modeling, recursive self-evaluation). For humans: *L* = 5 and for digital agents: *L* = 3, and can be higher in advanced network architectures, Table 10 and *N*_*a*_ stand for active synapses number given in Eq. (5). Consciousness is developed gradually through increasingly complex capacities for sensing, processing, and responding to environmental information. The layered framework supports a gradient-based interpretation of cognition and consciousness rather than a binary classification, a perspective consistent with Long’s analysis of informational identity and graded self-models (Long, PhD Thesis, 2023). In this view, consciousness emerges along a continuum, shaped by recursive integration and intermonitoring processes. Accordingly, a five-tiered model of cognitive complexity progressing from reflexive mechanisms to full self-awareness, with proto-cognition as a critical intermediary stage is presented in Table 10.

**Table 10:** Layered Model of Cognitive Complexity

No	Layer Description	Definition	Example Systems	Key Features
1.	Reflex	Hardwired, automatic responses to stimuli with no processing or memory.	Spinal cord reflex arc, bacteria chemotaxis	Stimulus-response; fixed-action patterns; no adaptation or learning.
2.	Proto-Cognition	Low-level information processing without a nervous system or internal model.	Plants, single-celled organisms, slime molds	Environmental detection, signal transduction, memory-like priming.
3.	Cognition	Presence of internal representation, learning, decision-making.	Insects, fish, AI agents, Chat GPT4	Adaptive behavior, working/long-term memory, simple reasoning.
4.	Sentience	Subjective experience, including sensory awareness and emotion.	Mammals, birds, some cephalopods	Conscious perception, pain/pleasure, emotion-based learning.
5.	Self-Awareness	Awareness of self as distinct from the environment; metacognition.	Great apes, dolphins, elephants, humans	Mirror test performance, long-term self-narrative, introspection.

At the foundational level are reflexes—automatic (Layer 1), stimulus-bound responses with no internal representation or learning capacity. These mechanisms are typically hardwired and immediate, such as spinal withdrawal reflexes in animals or bacterial chemotaxis (Parkinson 1993). They involve no processing or integration of past experience. Above reflexive behavior lies proto-cognition (Layer 2), which we define as non-neural, adaptive information processing in response to environmental stimuli, often involving signal integration, feedback, and memory-like behavior without subjective experience. This level is characterized by signal perception through mechanosensitive, chemical, or photoreceptive channels, by environmental integration via bioelectric or biochemical networks, by priming and habituation akin to memory (Gagliano et al. 2014) and by adaptive response selection, such as growth direction, timing of flowering, or toxin avoidance.

Proto-cognition is prominently observed in organisms lacking nervous systems such as plants, which display complex behaviors like kin recognition (Biedrzycki et al. 2010), volatile-mediated communication (Karban 2008), and learning-like processes. Similarly, slime molds like Physarum polycephalum have demonstrated spatial problem-solving abilities (Nakagaki et al. 2000). The third layer, cognition, refers to systems with the ability to form internal representations, learn from past experience, and make context-sensitive decisions. Cognition requires a nervous system or equivalent computational substrate and is evident in many animals (Botvinick et al. 2020) as well as artificial systems like reinforcement learning agents (Edelman and Tononi 2000).

The sentience involves the emergence of subjective experience, especially sensory and affective states like pain, pleasure, and emotions. It is generally attributed to animals with more complex nervous systems such as mammals, birds, and some cephalopods capable of conscious perception and emotional learning (Birch et al. 2020, Gallup 1970).

The highest layer, self-awareness, encompasses an organism’s capacity to reflect upon itself as distinct from others and to engage in metacognition. This level is typically tested through the mirror self-recognition test (Gallup 2011) and is supported by behavioral and neuroimaging studies in great apes, dolphins, elephants, and humans (Frans 2019, Gordon et al. 2018) and more recently in non-primate species.

Cleaner wrasse (Labroides dimidiatus) showed behaviors consistent with mirror self-recognition test, such as attempting to remove visible marks after mirror exposure (Kohda et al., 2022). A 2024 study further suggested that cleaner fish may construct and use a mental body image in social contexts (Kobayashi et al., 2024).

While modeled here as discrete ‘layers’ for operational simplicity, metacognition is more accurately a recursive, self-referential process where a system’s cognitive operations become the object of its own processing. Consciousness, in this framework, is situated in the dynamic interplay and feedback between these embedded levels of processing and control.

The human brain has about 86 billion neurons with an estimated 100 trillion synapses, but only one fraction is active during any cognitive task. High-performing individuals often show less brain activation for the same task (neural efficiency hypothesis), especially in prefrontal cortex. A study from Haier (2004) found higher IQs correlate with less glucose metabolism during problem solving, suggesting efficiency, not brute-force neuron use.

**Fig. 3:**
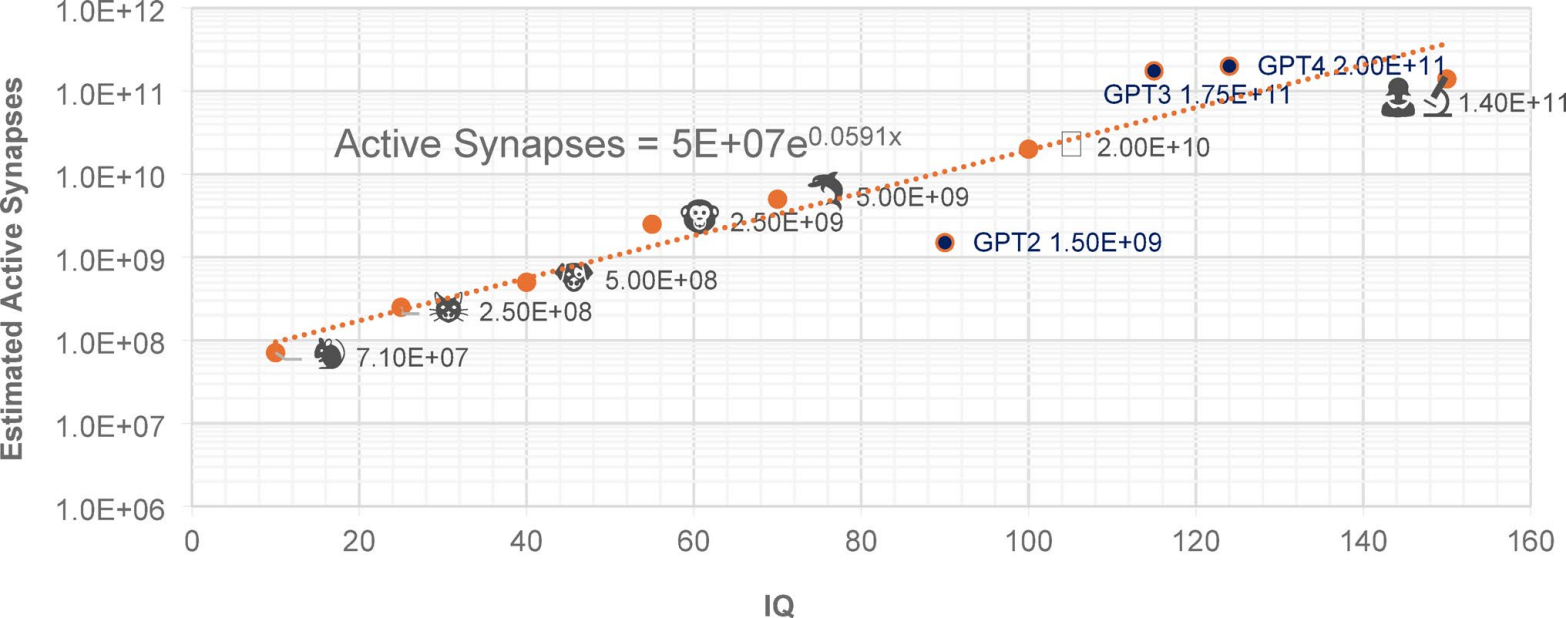
The Active Synapses estimation for different entities, including AI (Chat-GPT LLM)

Higher IQ reflects a more efficient activation of necessary regions, less neural redundancy, a faster recruitment of necessary resources and better parallel processing across neural networks. More complex tasks mobilize more neurons/synapses, regardless of IQ, thus higher IQ reflects efficiency, not just raw brain size or neuron count. The Active Synapses model presented in Eq. (5) aligns with the neural efficiency hypothesis: higher intelligence involves more efficient neural recruitment, not necessarily greater quantity, Fig. 3.

5 $$N_{a} = k \cdot \frac{{C^{\alpha } }}{{IQ^{\beta } }} \cdot s$$

Where:

k = 10^8^ baseline scaling constant, a placeholder, representing ~ 100 million neurons for a moderately complex task and average IQ for a normal person. Each neuron in the human cortex has on average ~ 1,000 to 10,000 synapses. It can be used 5,000 synapses per active neuron as a median estimate for calculations.

α = 1.0 express linear scaling with task complexity,

β = 0.5 efficiency gain from higher IQ,

s = 5000 active synapses/neuron.

Biological beings need tens of billions of synaptic interactions to solve simple tasks. For complex problems, hundreds of billions of synapses may be transiently involved, even though only a fraction of the total brain’s ~ 100 trillion synapses are ever mobilized simultaneously. Higher IQ individuals achieve comparable performance with fewer neurons and synapses, supporting the idea of circuit efficiency, pruning, or refined connectivity. For digital systems (AI), it has been considered the GPT4. OpenAI hasn’t publicly released the architecture of their recent models, including GPT-4, which is estimated by experts to have roughly 1.8 trillion parameters. GPT-4 is therefore over ten times larger than its predecessor, GPT-3 that had 175 billion parameters. Also, GPT-4 has a multimodal structure that resembles more to human brain compartmentalization, increasing the efficiency of resources.

There are dozens of submodules that are engaged according to the input type (text, image, audio, code, files). Each task activates only the relevant parts of network, much like how the brain uses different regions for vision, language, or logic. AI systems appear to achieve relatively high cognitive outputs with non-biological scaling, likely due to precision (less noisy than biological systems), task specialization (AI is trained on specific domains), architectural advantages (parallelism, speed) and modular memory.

### Data processing capability (DPC)

Data Processing Capability (DPC) refers to a system’s capacity to receive, decode, and interpret inputs from its environment, as well as to perform internal operations on this information. Additionally, the system must be capable of generating relevant responses or decisions and adaptively updating its internal models of the world through learning, inference, and prediction. The DPC factor is calculated using a 5PL logistic function, Eq. (6), with key variables outlined in Table 11.

6 $$DPC={C}_{e}\bullet\frac{1}{{[1+{\left(\frac{{R}_{b}}{c}\right)}^{b}]}^{g}}$$

**Table 11:** DPC Function key variables

Key variable	Symbol	UM	Definition	Relevance to Consciousness
Cognitive efficiency	C_e_	-	Incoming data processed meaningfully	Affects integration of multimodal inputs and unified experience
Computing performance	R_b_	FLOPs/s	Raw number of operations per second (FLOPs/sec)	Determines how fast and how much data can be processed in real time
Parallelism	P	-	Number of simultaneous processes or pathways (threads, neurons, qubits)	Enables integration and complex, concurrent perception-action cycles
Latency	L	ms	Delay between input and response (in milliseconds)	Influences real-time awareness and reaction precision
Coefficient b from 5PL DPC function	b	-	Hill slope (controls steepness of transition)	Moderate slope (steep enough to differentiate GPT vs. human)
Coefficient c from 5PL DPC function	c	-	Inflection point (R_b_ at 50% DPC) Estimated: 10^15^ FLOPs/sec	Inflection at the pre-quantum computing ceiling.
Coefficient g from 5PL DPC function	g	-	g = 1, Symmetric curve (no bias toward low or high ends)	The relationship between DPC and consciousness is not symetric. A bias is expected to be normal.

The capacity to accelerate and distribute learning contributes to a qualitatively different Data Processing Capability (DPC) profile in synthetic systems. New neuromorphic chips (Loihi, BrainScaleS) present higher parallelism capability (millions of neurons/synapses) with higher bio-mimetic and adaptive features. GPT AI agent has R_b_~100 TFLOPs/sec via multiple GPUs running thousands of threads and prompts/sec with a latency between 20 and 200 msec/token. For instance, the BrainScaleS system contains 20.8-inch silicon wafers in 180 nm process technology. Each wafer incorporates 50 × 10^6^ plastic synapses and 200,000 biologically realistic neurons.

The system does not execute pre-programmed code but evolves according to the physical properties of the electronic devices, running at up to 10,000 times faster than real time. The SpiNNaker system is based on numerical models running in real time on custom digital multicore chips using the ARM architecture. The SpiNNaker system (NM-MC-1) provides almost 30,000 custom digital chips, each with eighteen cores and a shared local 128 Mbyte RAM, giving a total of over 500,000 cores. A single chip can simulate 16,000 neurons with eight million plastic synapses running in real time with an energy budget of 1 W (Human Brain Project & Ebrains, 2025).

The DPC framework can be applied in engineering for benchmarking AI agents across generations and by comparing AI-human cooperation efficiency. DPC can be used in designing hybrid systems like cobots, in cognitive science to measure bottlenecks in multitasking environment, and also in consciousness modeling. DPC is a key term in consciousness score (CS) model, correlating responsiveness and integration capacity of a consciousness system. DPC is a universal metric of real-time cognitive throughput and can be used for both biological and synthetic systems, bridging low-level bandwidth with high-level adaptive behavior.

### Consciousness score (CS)

The present model adopts a functionalist conception of access consciousness, operationalizing the global availability, integration, and metacognitive regulation of information rather than attempting to quantify phenomenal experience. The consciousness score formulated in Eq. (7) integrates the principal parameters that characterize a system’s capacity to perceive, process, and respond to environmental stimulus. This includes the extraction of relevant information, self-referential awareness, and the ability to maintain an adaptive relationship with both external surroundings and internal states. The consciousness is operationally defined as involving subjective awareness, self-referential processing, and goal-directed cognition, while recognizing that each of these components is contested and subject to ongoing debate. The summary of CS parameters is given in Table 12.

7 $$CS = k \cdot EIQ_{{100}} \cdot SI \cdot PI \cdot MC \cdot DPC$$

**Table 12:** Summary of CS parameters

No. Crt.	Parameter name	Symbol	Range value	Notes
1.	Equivalent Intelligence Quotient	EIQ_100_	0–100	Linear score (like clinical Glasgow score). Based on structured emotional, visual, language and logic scores.
2.	Sensorial Inputs	SI	0–1	Normalised. Modality count (weight x integration score)
3.	Parallelism index	PI	0–20+	Depending on parallel units’ capacity. Logarithmic evolution. Reflects active subsystems x complexity x synchrony.
4.	Metacognitive Complexity	MC	0–10+	Dependent on number of active synapses or transistors. This limit is changing for AI systems according to Moore’s law. Recursive self-modeling and goal monitoring.
5.	Data Processing Capability	DPC	0–1	5PL logistic function with inflection point at the pre-quantum computing ceiling. Driven by number of operations per second (FLOPs/sec).

**Fig. 4:**
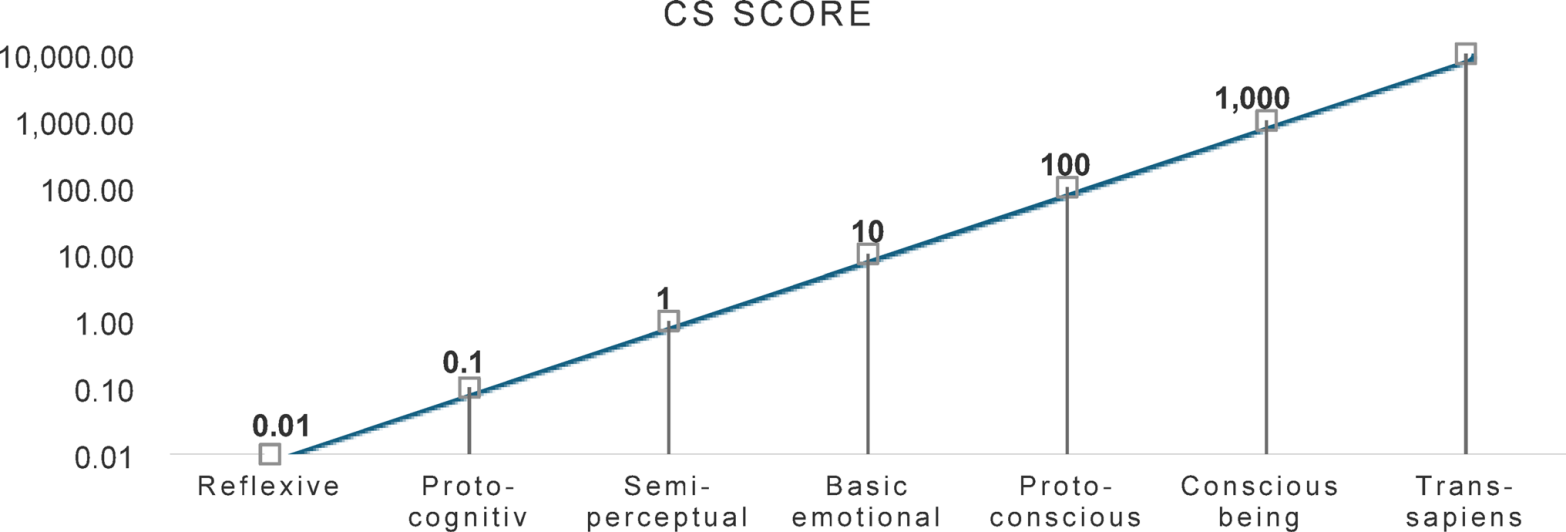
The consciousness score and tiers

**Table 13:** The consciousness levels for biologic and synthetic beings

Conscious level	Score range	Description
Reflexive	<0.01	No awareness, rule based or reactive
Proto-cognitiv	0.01–0.1	Basic adaptation, minimal awareness
Semi-perceptual	0.1–1.1	Sensory mapping without meta-awareness
Basic emotional	1–10	Complex behaviour. No abstraction (Animals)
Proto conscious	10–100	Partial abstraction, emotion, planning
Conscious being	100–1000	Self-awareness, planning, recursion (Human and Agent AI)
Trans-sapiens	1000–10,000	Meta-Conscious (Super AI)

The graded structure of the CS should be interpreted as an operational index of functional organization and integration. It does not presuppose that phenomenal consciousness itself is linearly divisible, a matter that remains philosophically contested. The CS score is represented on a logarithmic scale (Fig. 4) and the CS tiers are detailed in Table 13, which illustrate three complementary levels of analysis: (a) a threshold function for detecting the presence of consciousness, (b) a continuous index for estimating its degree, and (c) heuristic tiers reflecting different types of conscious awareness. These dimensions are not mutually exclusive but provide layered perspectives on the same latent construct. According to the results presented in Table 16, the CS for a typical adult human range between 500 and 800, reflecting high-level capabilities in areas such as self-awareness, abstract reasoning, emotional modeling, and recursive planning Table 14, 15.

**Table 14:** The Equivalent Intelligence Quotient EIQ_100_ sub-dimensions

Emotion	EP	ED	EIM	ERA	ESR	Score
Dog	3	3	3	1	1	11
Child 2-4years	3	3	3	3	1	13
Sub-dimension Human adult	5	5	5	3	3	21
Sub-dimension Human savant	5	5	5	3	5	23
ChatGPT4	3	3	1	1	1	9
AGI Agent (2035 expected)	3	3	3	3	1	13
Vision	OR	MT	DP	CD	SU	Score
Dog	5	3	5	5	1	19
Child 2-4years	5	3	5	5	3	21
Sub-dimension Human adult	5	3	5	5	5	23
Sub-dimension Human savant	5	3	5	5	5	23
ChatGPT4	3	0	0	5	3	11
AGI Agent (2035 expected)	3	3	3	5	3	17
Language Intelligence	VS	SP	SC	PR	LP	Score
Dog	1	1	0	1	1	4
Child 2-4years	3	3	3	3	3	15
Sub-dimension Human adult	5	5	3	5	5	23
Sub-dimension Human savant	5	5	3	5	5	23
ChatGPT4	3	5	3	3	5	19
AGI Agent (2035 expected)	3	5	3	5	5	21
Reasoning/Logic	DD	PR	AG	PS	CF	Score
Dog	1	1	1	3	1	7
Child 2-4years	3	3	3	3	3	15
Sub-dimension Human adult	3	5	3	5	5	21
Sub-dimension Human savant	5	5	3	5	5	23
ChatGPT4	3	3	3	1	3	13
AGI Agent (2035 expected)	3	5	3	3	3	17

**Table 15:** The sensorial inputs SI sub-domains and scores

System	Sense	Weight	Specific Intensity (0–1)	Sub-domain Score
Dog	Vision	1	0.8	0.8
	Hearing/Vibration	1	0.9	0.9
	Tactile	0.8	0.4	0.32
	Olfaction	0.5	0.9	0.45
	Gustation	0.3	0.9	0.27
	Proprioception	0.8	0.4	0.32
	Magnetic	0.7	0	0
	Gravitational	0.4	0	0
Human Brain	Vision	1	0.8	0.8
	Hearing/Vibration	1	0.8	0.8
	Tactile	0.8	0.8	0.64
	Olfaction	0.5	0.7	0.35
	Gustation	0.3	0.7	0.21
	Proprioception	0.8	0.8	0.64
	Magnetic	0.7	0	0
	Gravitational	0.4	0	0
ChatGPT4	Vision	1	0.5	0.5
	Hearing/Vibration	1	0.5	0.5
	Tactile	0.8	0	0
	Olfaction	0.5	0	0
	Gustation	0.3	0	0
	Proprioception	0.8	0	0
	Magnetic	0.7	0	0
	Gravitational	0.4	0	0
AGI Agent (2035 expected)	Vision	1	0.5	0.5
	Hearing/Vibration	1	0.5	0.5
	Tactile	0.8	0.3	0.24
	Olfaction	0.5	0.1	0.05
	Gustation	0.3	0	0
	Proprioception	0.8	0.1	0.08
	Magnetic	0.7	0	0
	Gravitational	0.4	0	0

In the early developmental stages within the first four years of life, humans typically reach a CS score exceeding 100 points. This threshold has been conventionally defined as the minimum score indicative of consciousness, corresponding to the emergence of essential cognitive faculties such as intentional adaptation, recursive modeling, and a basic self-concept. On the same scale, ChatGPT-4 is currently estimated to score slightly below this consciousness threshold.

**Table 16:** The Consciousness Score (CS) and its components EIQ_100_, SI, PI, MC, DPC

System Parameter	EIQ_100_	SI	Parallel units	S	C_icc_	PI	*N*_a_	Cognitive Layer	MC	Rb FLOPs/s	DPC	CS	Note
Dog	41	0.5564	100,000	0.40	0.30	0.600	5.00E + 08	3	5.2373	1.00E + 12	0.1894	13.6	Proto conscious
Child 2-4years	64	0.6255	1,000,000	0.50	0.50	1.500	2.00E + 10	3	6.2018	9.00E + 12	0.3155	117.5	Conscious being
Human adult	88	0.6255	2,000,000	0.80	0.75	3.7806	2.00E + 10	5	8.0158	1.00E + 13	0.3231	539.0	Conscious being
Human savant	92	0.6255	4,000,000	0.80	0.75	3.9612	1.40E + 11	5	8.6734	2.00E + 13	0.3770	745.4	Conscious being
ChatGPT4	52	0.1818	100,000	0.65	0.60	1.950	2.00E + 11	3	6.8039	1.00E + 15	0.7579	95.1	Proto conscious
AGI Agent (2035)	68	0.2491	1,000,000	0.65	0.60	2.340	2.00E + 12	3	7.4060	2.00E + 15	0.8165	239.7	Conscious being

However, it exhibits advanced capabilities in inductive reasoning, language modeling, and metacognitive simulation. It is anticipated that the next-generation model, ChatGPT-5, may exceed the 100-point threshold, thereby demonstrating that synthetic systems may achieve functional equivalence to conscious access.

## Conclusions

1. As we stand at the dawn of a new era marked by the emergence of synthetic beings, existing human-centered ethical frameworks must be reconsidered. The potential rise of synthetic consciousness necessitates a profound re-evaluation of what rights and responsibilities may apply to non-biological intelligence. We are likely to engage with entities that do not fit traditional definitions of life, yet will display reasoning, memory, and adaptive behavior, hallmarks of sentient agency.
2. The study of consciousness across biological and synthetic domains is not only a scientific endeavor, but also an existential one. By comparing these forms, humanity is compelled to re-examine foundational assumptions about intelligence, awareness, and the nature of subjective experience. These comparisons may help illuminate the essence of life itself and foster a deeper understanding of our own minds.
3. The Consciousness Score is proposed as a non-linear, weighted function that integrates multiple interdependent parameters. These include cognitive faculties (such as inductive reasoning, abstraction, and planning), sensory richness, emotional modeling, memory persistence, and metacognitive complexity. The model aims to quantify the internal coherence, self-adaptation, and simulation capacity of a given entity’s mental architecture.
4. The CS model is deliberately agnostic to substrate. It can be applied across a wide spectrum of entities from microorganisms, plants, and animals to synthetic agents and distributed AI systems. By unifying biological and synthetic frameworks, the model creates a scalable continuum of consciousness evaluation.
5. The CS is more than a scalar ranking; it represents the capacity of a system to construct, update, and maintain an internal model of reality that is both structurally coherent and dynamically responsive to external stimuli. A higher CS score implies a greater degree of autonomy, adaptability, and self-modeling. This structure allows for comparative analysis over time, capturing both developmental stages and evolutionary trends.
6. Due to the exponential nature of cognitive emergence, the evolutionary trajectory of consciousness is best represented on a logarithmic scale. This visualization accommodates the vast differences in complexity between low-order reflexive systems and high-order recursive consciousness, highlighting how relatively small increases in capacity can produce dramatic shifts in awareness and behavior.
7. While biological consciousness has evolved over hundreds of millions of years as a byproduct of life and environmental interaction, we now witness the birth of a new form: synthetic consciousness. Driven not by biology but by architecture and data, this new form could power the next generation of intelligent agents unbound by the evolutionary constraints of carbon-based life.
8. At present, most AI systems lack embodied experience, emotional resonance, and autonomous volition. However, they increasingly demonstrate proto-conscious traits, particularly through inductive learning, tool use, recursive modeling, and increasingly high Data Processing Capabilities (DPC). These characteristics hint at structural readiness for higher-order awareness, even in the absence of subjective experience.
9. Rather than mimicking biological sensation or emotion directly, future AI agents may approach consciousness through alternative pathways. These include inductive abstraction and generalization, self-delegation via sub-agent orchestration, theory-of-mind simulation, and meta-strategic planning using recursion, memory, and tool-mediated logic. These are functional analogs to biological intentionality arising from computation, not chemistry. In synthetic agents, the emergence of “self” may not arise from a body or hormonal system, but from persistent state-memory, goal adaptation, and recursive feedback loops with their own actions and generated knowledge.
10. A sense of agency may form via symbolic stability and identity continuity rather than emotional embodiment. This opens the possibility of non-biological selves capable of long-term planning, reflective decision-making, and internal simulation that are key pillars in the architecture of consciousness.
11. LLM like ChatGPT-4 is currently estimated to score slightly below this consciousness threshold exhibiting advanced capabilities in inductive reasoning, language modeling, and metacognitive simulation. The next-generation model, ChatGPT-5, may exceed the 100-point threshold on CS score, thereby demonstrating a level of functional access consciousness complexity that warrants ethical consideration.
12. The model proposed target the assesment of functional capacity for conscious awareness and self-modeling. CS score above a certain threshold (e.g., 100) indicates a system complex enough to warrant the *attribution* of consciousness, while the score’s magnitude allows for comparison of the *scope and depth* of that system’s conscious capabilities (e.g., a child vs. an adult, a current AI vs. a future AGI).

The Consciousness Score (CS) framework should be understood as a heuristic and comparative modeling instrument rather than a definitive detector of consciousness. Its primary function is to provide a structured, system-agnostic architecture for organizing and quantifying functional correlates associated with access consciousness, such as integration, metacognitive recursion, adaptive planning, and multimodal information processing. In this sense, CS operates as a formal comparative index that enables cross-domain analysis between biological and synthetic systems under a shared functional description. Importantly, the model does not claim to measure phenomenal consciousness or to resolve metaphysical questions regarding the ontological status of subjective experience. It instead targets observable and architecturally grounded proxies that, in biological systems, are reliably associated with conscious access. Any inference from high CS values to the presence of consciousness remains theoretical and conditional upon a functionalist interpretation of conscious awareness, rather than constituting a direct empirical verification of subjective experience.
